# Prediction of Drug-Target Interactions for Drug Repositioning Only Based on Genomic Expression Similarity

**DOI:** 10.1371/journal.pcbi.1003315

**Published:** 2013-11-07

**Authors:** Kejian Wang, Jiazhi Sun, Shufeng Zhou, Chunling Wan, Shengying Qin, Can Li, Lin He, Lun Yang

**Affiliations:** 1 Bio-X Institutes, Key Laboratory for the Genetics of Developmental and Neuropsychiatric Disorders, Shanghai Jiao Tong University, Shanghai, China; 2 Department of Pharmaceutical Sciences, College of Pharmacy, University of South Florida, Tampa, Florida, United States of America; Accelrys, United States of America

## Abstract

Small drug molecules usually bind to multiple protein targets or even unintended off-targets. Such drug promiscuity has often led to unwanted or unexplained drug reactions, resulting in side effects or drug repositioning opportunities. So it is always an important issue in pharmacology to identify potential drug-target interactions (DTI). However, DTI discovery by experiment remains a challenging task, due to high expense of time and resources. Many computational methods are therefore developed to predict DTI with high throughput biological and clinical data. Here, we initiatively demonstrate that the on-target and off-target effects could be characterized by drug-induced *in vitro* genomic expression changes, e.g. the data in Connectivity Map (CMap). Thus, unknown ligands of a certain target can be found from the compounds showing high gene-expression similarity to the known ligands. Then to clarify the detailed practice of CMap based DTI prediction, we objectively evaluate how well each target is characterized by CMap. The results suggest that (1) some targets are better characterized than others, so the prediction models specific to these well characterized targets would be more accurate and reliable; (2) in some cases, a family of ligands for the same target tend to interact with common off-targets, which may help increase the efficiency of DTI discovery and explain the mechanisms of complicated drug actions. In the present study, CMap expression similarity is proposed as a novel indicator of drug-target interactions. The detailed strategies of improving data quality by decreasing the batch effect and building prediction models are also effectively established. We believe the success in CMap can be further translated into other public and commercial data of genomic expression, thus increasing research productivity towards valid drug repositioning and minimal side effects.

## Author Summary

Small drug molecules usually bind to unintended off-targets, leading to unexpected drug responses such as side effects or drug repositioning opportunities. Thus, identifying unintended drug-target interactions (DTI) is particularly required for understanding complicated drug actions. It remains expensive nowadays to experimentally determine DTI, so various computational methods are developed. In this study, we initiatively demonstrated that target binding is directly correlated with drug induced genomic expression profiles in Connectivity Map (CMap). By improving data quality of CMap, we illustrated three important facts: (1) Drugs binding to common targets show higher gene-expression similarity than random compounds, indicating that upstream ligand binding could be characterized by downstream gene-expression change. (2) It is found that some targets are better characterized by CMap than others. To guarantee efficiency of DTI discovery, prediction models should be specifically built for those well characterized targets. (3) It is broadly observed in the predicted DTI that ligands for the same target may collectively interact with common off-target. This observation is consistent with published experimental evidence and can help illustrate the mechanisms of unexplained drug reactions. Based on CMap, our work established an efficient pipeline of identifying potential DTI. By extending the success in CMap to other genomic data sources, we believe more DTI would be discovered.

## Introduction

Drug promiscuity refers to the phenomenon that small molecule drug binds to multiple protein targets. In recent years, drug promiscuity has gained broad attention [1]–[3], because unintended drugs-target interactions (DTI) are often associated with drug repositioning [4] and side effects [5]–[8]. Although biotechnology evolves and new biochemical assays arise [9], [10], it remains time-consuming and expensive nowadays to experimentally discover unknown DTI, especially when multiple compounds and proteins are simultaneously involved. This situation therefore provides a strong incentive to develop new computational methods, which could screen potential DTI with high throughput and low cost.

**Figure 1:**
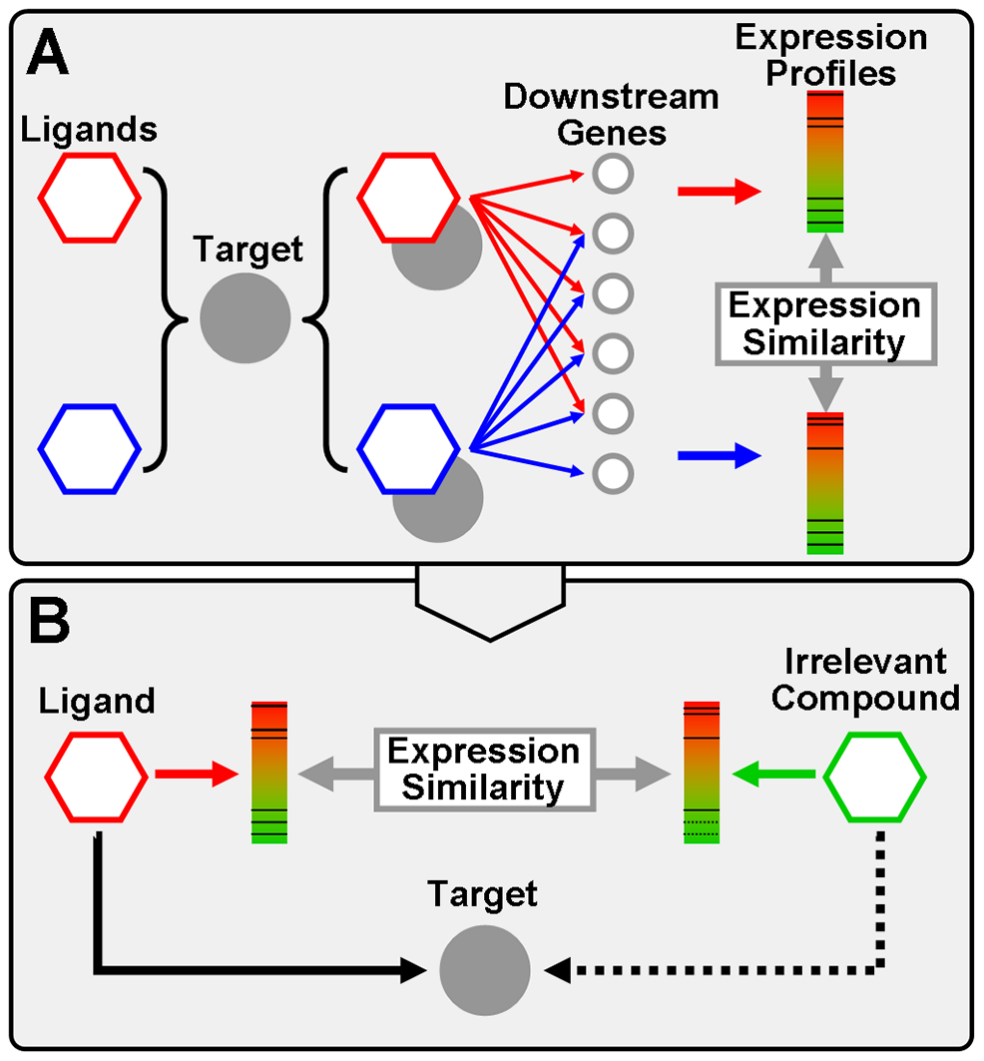
The principle of DTI prediction based on gene-expression information. (A) Ligand-binding modifies the biological functions of protein target, a series of target-related downstream genes are then influenced. Thus, we suppose that variant ligands binding to the same target should influence some downstream genes in common. This hypothesis is corroborated by the fact that drugs sharing common targets result in similar gene-expression profiles in CMap. (B) We therefore applied CMap expression similarity as a guilt-by-association indicator of potential drug-target interactions. If one compound has no recognized interaction with one certain target but shows high expression similarity to the ligands of that target, it may imply undiscovered drug-target interaction.

By binding to targets with complementary structures, drug molecules profoundly modify the behavior of downstream genes and lead to specific reactions. Along this route of drug action, various biological informations could be correlated to target binding and be analyzed with computational models. For example, methods have been established to predict DTI by ligand/protein structures [11]–[16] and clinical side effects [17]. On the other hand, although there are researches addressing drug-induced target expression [18], it has been rarely studied that drug-induced downstream gene-expression changes may directly indicate target promiscuity, thus missing a possible technique of DTI discovery. Here we suppose that drugs binding to specific target are generally prone to influencing the target-related downstream genes [19], [20], so the pattern of gene-expression change could reflect the characteristics of target binding (Figure 1A). One of the most reliable and comprehensive sources of drug-induced genomic expression data is the Connectivity Map (CMap), which includes 6100 human cell cultures (i.e. 6100 CMap ‘instances’) treated by 1309 bioactive compounds [21]. We initiatively found that drugs interacting with the same target generally lead to similar gene-expression profiles in CMap. This observation enlightened us to apply CMap expression similarity as a guilt-by-association metric, that high similarity between different drugs may imply interactions to the same target (Figure 1B)

However, one of the major impediments of CMap data analysis is so-called ‘batch effect’ [22], i.e. cells under the same culture condition lead to highly similar expression patterns, even if they are treated by totally different compounds. In order to overcome the batch effect and make CMap data reflect more signal than noise, a variety of new protocols are successively developed [18], [22]–[24], suggesting the importance of this issue. In order to adjust batch effect as well as keep the integrity of CMap data, we implemented here a novel method to bridge the gap between different batches upon homogeneous drug treatments. Comparing adjusted data with original CMap, we saw that our adjustment procedures lead to improved efficiency of connecting drugs with common protein targets, which solidly facilitated the discovery of potential DTI.

## Results/Discussion

### Adjusting the batch effect in CMap data

**Figure 2:**
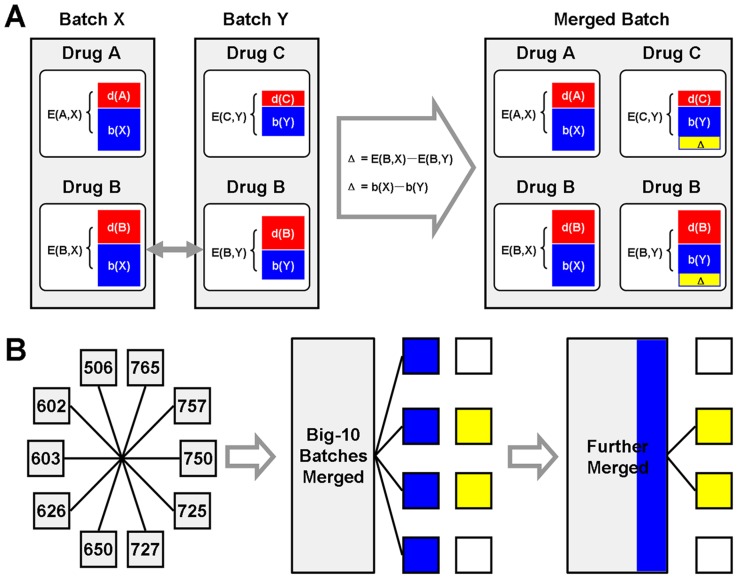
The rationale of batch effect adjustment. (A) The expression profile (denoted as variable E) in CMap is mainly determined by drug action (component d) and batch effect (component b). While the cell condition may vary from batch to batch, the drug action is relatively consistent. Thus, if batch X and Y include cell cultures treated by the same drug (e.g. drug B), these two drug B related expression profiles reflect homogenous drug action but heterogeneous cell condition. So their difference (denoted as Δ) reflects the variation between batch X and Y. By adding Δ to expression profiles in batch Y, the batch variation is adjusted and the two batches are merged into one. (B) Among the batches with 30 instances or more, we find 10 of them linking to each other by various bridge drugs. Primarily, we merged these 10 batches into a new one. Then other batches sharing bridge drugs with this new batch are further merged to form an even bigger batch. This bridging procedure is repeated until all batches are adjusted.

In order to accurately predict DTI with gene-expression profiles, we primarily improved the reliability of CMap data. Ideally, the gene-expression profile of each CMap instance should be solely determined by the bioactivity of treating compound. But the signal is confounded by batch variation, which makes the gene-expression profiles of different batches much less comparable. Iskar et al. [18] used a ‘mean-centering’ method to remedy the batch effect, but at the cost of abandoning many instances in small batches. To present a complete evaluation of CMap based DTI discovery, we therefore developed a novel method that not only overcomes batch variation but also retains all instances (Text S1, Figure S1 and Table S1). We hypothesize that if two instances belonging to different batches are treated by the same drug, the drug action should be homogeneously reflected in two gene-expression profiles, so their difference should be mainly attributed to batch variation. Based on this hypothesis, we select the instances treated by the same drug as ‘bridges’ between two batches, so batch variation is estimated by the difference between bridge instances (see Methods). If the estimated quantity of batch variation is added to the original gene-expression profiles, two different batches could be, in a sense, regarded as derived from the same cell culture and merged into one (Figure 2A).

To bridge the batch variation across all CMap instances, we primarily selected 10 big batches (with not less than 30 instances each) that share a variety of bridge instances ( and Table S3). These 10 big batches are merged together, then other batches are further merged via bridges and so on (Figure 2B and Text S2). Finally, all 6100 instances of 302 batches are unified into an adjusted dataset (freely available upon request).

Across different cell lines and treatment dosages, the instances treated by the same compound are collectively considered, that the fold changes of gene-expression are averaged to obtain a single ‘synthetic expression profile’ for each compound. To measure the gene-expression similarity between two different compounds, we calculated the Bridge Adjusted Expression Similarity (BAES, see Methods) by using a protocol similar as the Gene Set Enrichment Analysis (GSEA) algorithm described in the original CMap publication [21]. In a total, 856,086 BAES scores were calculated across all 1309 CMap compounds. In the same way, we also calculated the gene-expression similarity for original (unadjusted) CMap data.

### The correlation between DTI and gene-expression profiles

We assess the efficacy of CMap adjustment by evaluating the correlation between BAES and well-known drug-target interactions. DrugBank database, so far, is one of the most acknowledged sources of drug target information [25]. We therefore mapped the drugs enrolled in DrugBank to the CMap compounds, obtaining 2084 interactions between CMap compounds and 731 DrugBank targets.

**Figure 3:**
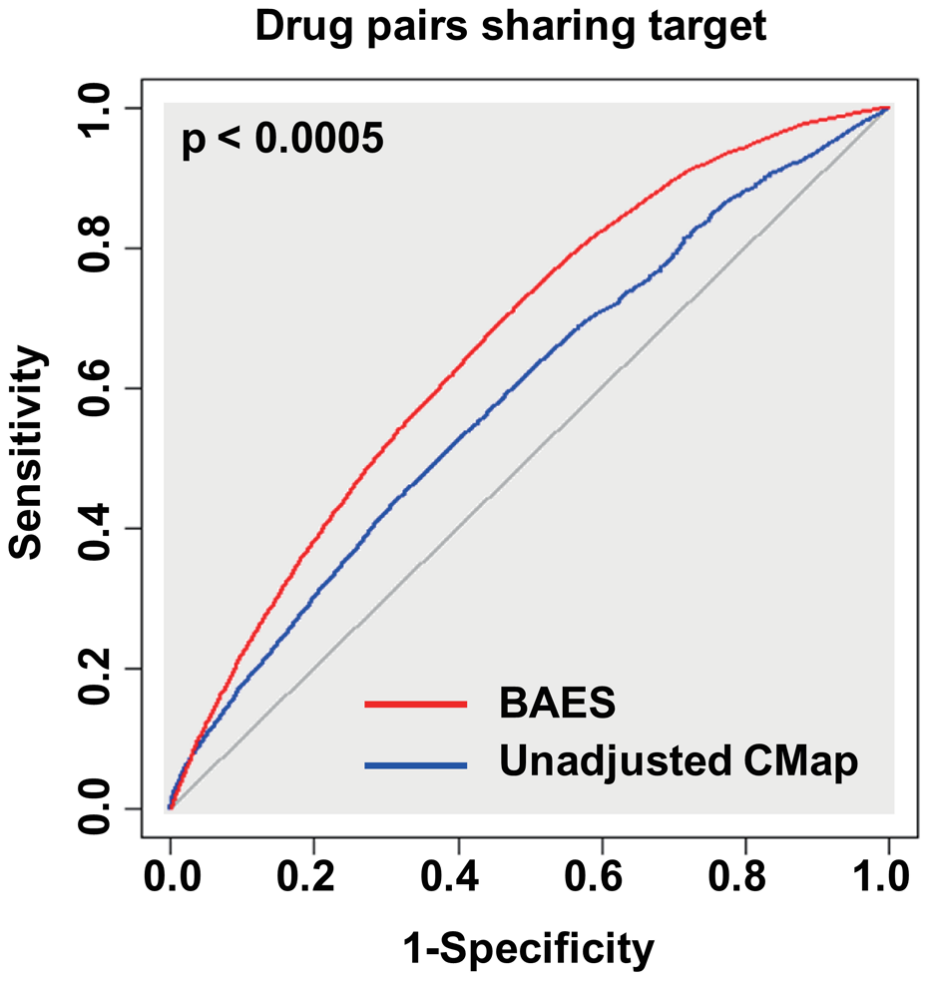
Receiver operating characteristic (ROC) curve is used to evaluate the performance of BAES score and unadjusted CMap expression similarity. For the classification between compound pairs sharing target (positive set) or not (negative set), the area under curve for BAES and unadjusted CMap is 0.66 and 0.59, respectively. The advantage of BAES is verified with 2000 replicates of bootstrap test, by the pROC package for R (http://cran.r-project.org/web/packages/pROC/).

We expect that drugs binding to common target result in higher pairwise similarity in gene-expression profiles than random compounds. And it is observed that the BAES significantly outperforms the unadjusted expression similarity [26], in terms of scoring compound pairs that share at least one target in DrugBank (Figure 3). This test corroborates that after batch effect adjustment, CMap expression profiles would better characterize the genomic reactions of ligand binding. Thus, BAES could be used as a guilt-by-association metric to detect potential drug-target interactions, that drugs show high BAES may interact to the same target.

### The efficiency of CMap based prediction models

**Figure 4:**
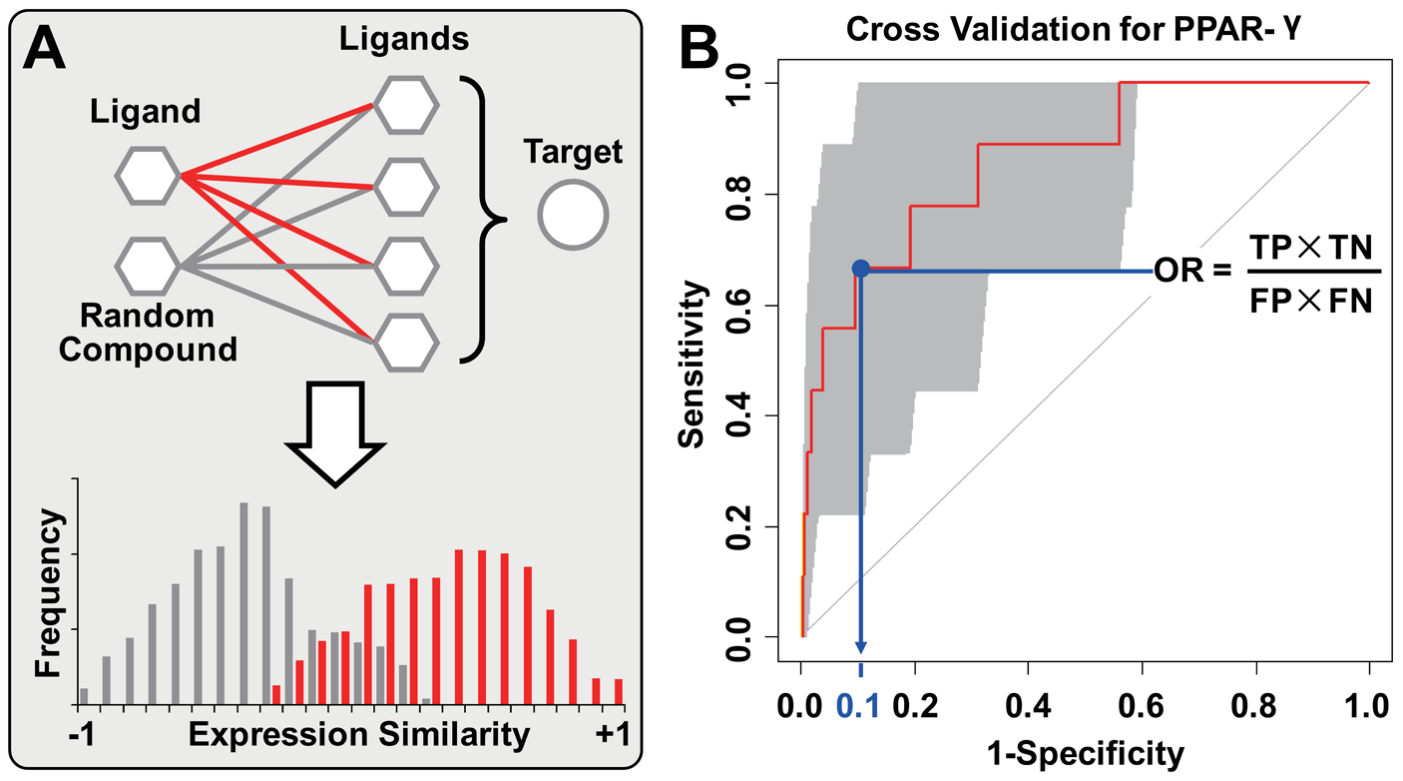
The rationale and performance of DTI prediction model. (A) If a target has its ligand-binding well characterized by CMap, we expect the potential ligands to show higher BAES to benchmark ligands (red colored connections) than random compounds do (grey colored connections), i.e. the LOI of ligands should excel the overall background of CMap. (B) For the cross validation of PPAR-γ, the area under ROC curve reaches 0.86, with the 95% confidence interval (i.e. the grey colored shape) ranging from 0.74 to 0.99. The LOI corresponding to 90 percent specificity is set as the threshold to discriminate positive and negative sets. Thus, only 10 percent of the negative set is above the threshold, i.e. there would be 130 false positive (FP) and 1170 true negative (TN) compounds. Meanwhile, 67 percent of the positive set is above the threshold, so there would be 6 true positive (TP) and 3 false negative (FN) compounds. The statistical significance of such enrichment (odds ratio = 18) is determined by Fisher's exact test (p = 7.31×10^−5^).

To demonstrate the genuine power of CMap based DTI prediction, we adopted a type of naïve model without any fitting process. For a given target, its designated ligands recorded in DrugBank are defined as ‘benchmarks’. Given the correlation between DTI and gene-expression similarity, we expect the true ligands to show higher BAES to benchmarks than random compounds do. Thus, the likelihood of DTI can be measured by the average BAES between a candidate compound and a series of benchmark ligands (Figure 4A), that higher BAES should indicate higher ‘likelihood of interaction’ (LOI, see Methods).

This model is applied to each human protein target, and the performance is evaluated with leave-one-out cross validation (LOOCV) (see Methods). Taking peroxisome proliferator-activated receptors gamma (PPAR-γ, encoded by PPARG gene) as an example, the 9 PPAR-γ ligands enrolled in CMap are set as benchmarks. All 1309 CMap compounds, including the benchmark ligands (positive set) and other compounds (negative set), are ranked by LOI in LOOCV. Two criteria are used to determine whether DTI is effectively characterized by BAES. Primarily, the area under receiver operating characteristic (ROC) curve should be high and robust. Additionally, the benchmark ligands should be particularly enriched in the drugs with high LOI, thus ensuring the practicability of detecting hidden ligands from the top-ranked drugs. We therefore calculated the 95% confidence interval of area under curve (AUC) [26] and the odds ratio of positive set enrichment. In the above example of PPAR-γ, we can see that most benchmark ligands of PPAR-γ show relatively high LOI, leading to robust ROC curve and significant enrichment of positive set (Figure 4B).

**Figure 5:**
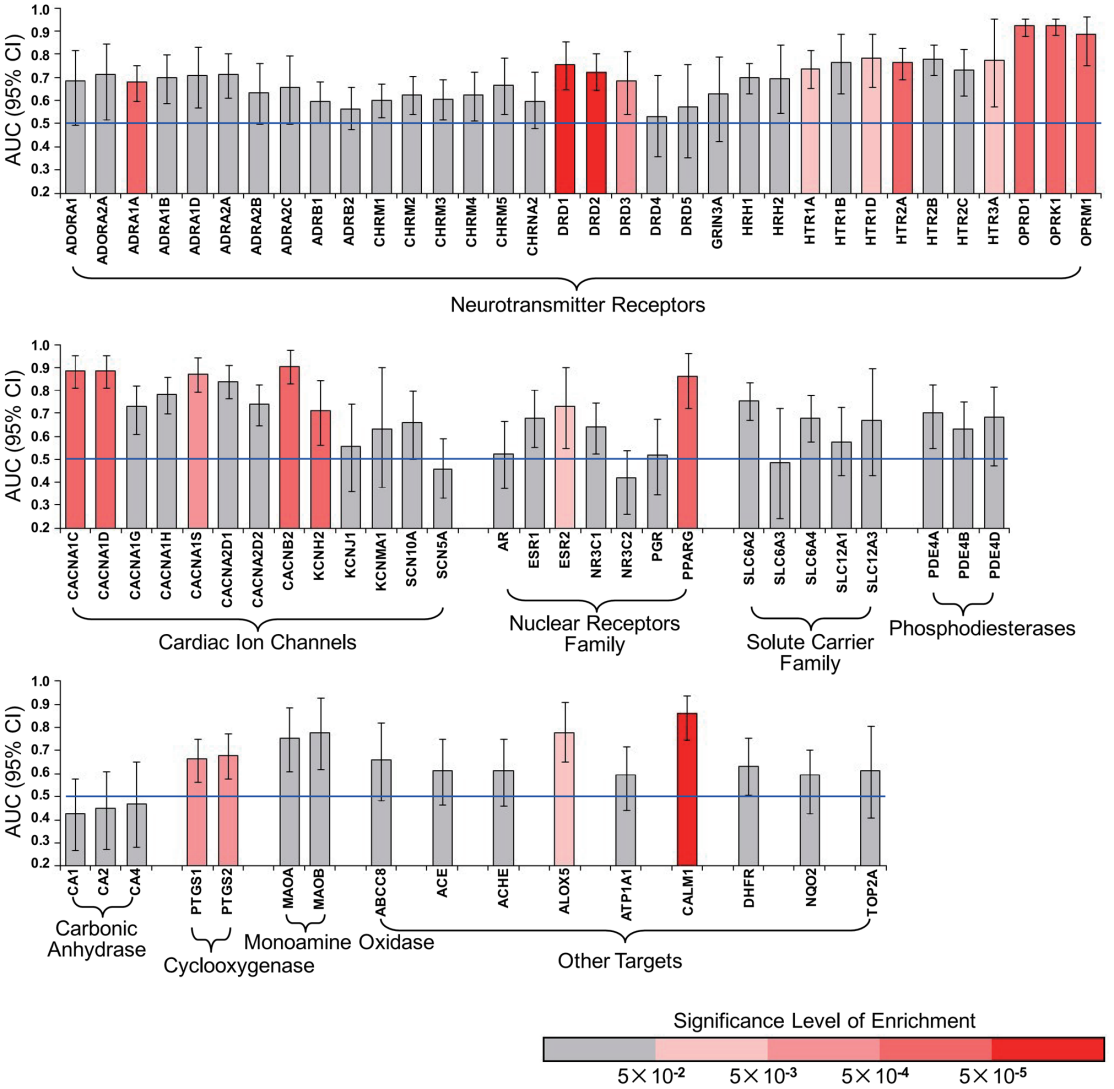
The performance of LOOCV suggests that at mRNA level, the genomic reactions of ligands binding differ dramatically from target to target. Here all the targets are displayed in several families, according to their functional origins. The height of each bar represents the AUC level (and 95% confidence interval). And the color of each bar indicates the significance level of benchmark ligands enrichment.

For most tested targets (72 out of 78, accounting for 92%), the benchmark ligands are distinguished from other CMap compounds (i.e. AUC>0.50), suggesting the general efficiency of BAES model. On the other hand, examining the robustness of ROC curve and the benchmark enrichment in top-ranked drugs, we find that individual targets are differentially characterized by CMap (Figure 5 and Data S1). The well characterized targets with robust ROC curve (i.e. the lower bound of AUC confidence interval is over 0.50) and significant benchmark enrichment (i.e. the p-value is less than 0.05) are more likely to be found among neurotransmitter receptors, ion channels, nuclear receptors and cyclooxygenases. Such distinction of performance indicates that the ligands binding of some targets, but not others, can particularly result in extensive and intensive changes at mRNA level, which is exactly detectable in CMap. So instead of building a universal model to predict interactions across all drugs and targets, we suggest that specified models should be established for individual targets (especially the targets well characterized by CMap), in order to increase the chance of detecting true DTI.

### Further attempts to improve DTI prediction

Besides the well characterized targets, we found that a variety of targets (such as some neurotransmitter receptors, several calcium ion channels and monoamine oxidases etc.) also exhibit high odds ratio of benchmark enrichment, but not high significance level (Data S1). These observations could be attributed to the limited number of enrolled benchmark ligands (i.e. a small positive set). As a result, the power of Fisher's exact test is impaired, due to too few true positive and false negative samples. For example, serotonin receptor HTR1B showed even better ROC curve than the well characterized target HTR1A, but could not pass the significance test (Figure S2).

This suggests that the sufficiency of benchmark ligands information is critical to the robustness and reliability of CMap based DTI prediction. We therefore look forward to translating the success in CMap into other large-scale genomic expression data resources (such as Gene Expression Omnibus [27] built by NCBI and classified data submitted to FDA by drug developers [28]) or high-throughput data derived from individual studies [29]. Since the data variation brought by the difference of experiment conditions can be effectively adjusted with appropriate computational methods (e.g. BAES), we believe that many external data could turn to be comparable to CMap profiles [30]. Then expression profiles of additional ligands (not enrolled in CMap) can be further used as benchmark ligands, in order to improve the DTI prediction models.

### From drug-target interactions to target-target interactions

**Figure 6:**
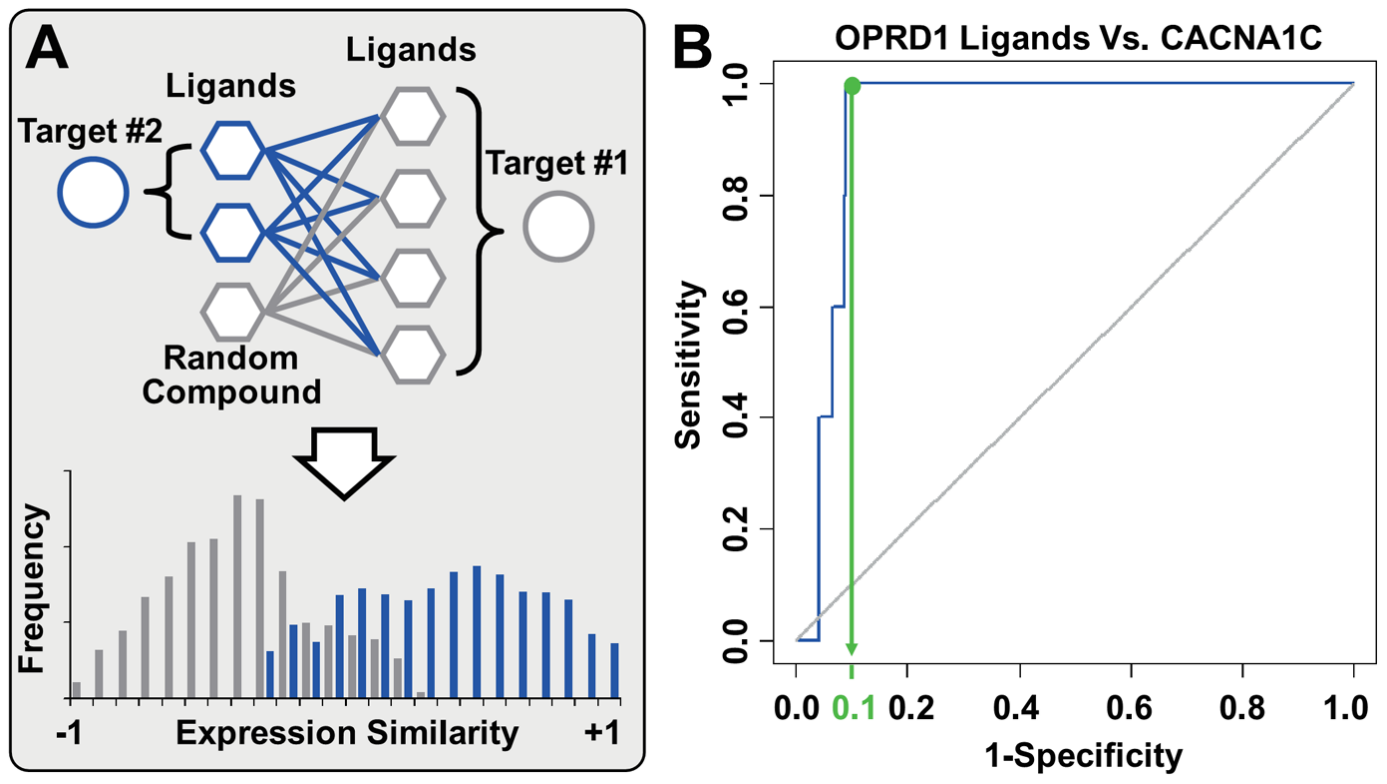
Identifying of target-target interactions by gene-expression similarity. (A) In some cases, the ligands of one target may generally show high LOI to another distant target. (B) The OPRD1 ligands (positive set) collectively show higher LOI to CACNA1C than other compounds (negative set). Setting the LOI corresponding to 90 percent specificity as threshold, we find that all OPRM1 ligands are above the threshold (p = 1.08×10^−5^).

By using the CMap based model, the compounds showing high LOI to particular target are identified, thus providing drug-target pairs with potential interactions. We notice that in some cases, the designated ligands of one certain target tend to collectively interact with another specific off-target (Figure 6A). For instance, the designated ligands of opioid receptor OPRD1 generally exhibit high LOI to calcium channel CACNA1C (Figure 6B), even if OPRD1 and CACNA1C have no DrugBank ligands in common (i.e. they are ‘distant targets’). This phenomenon indicates that the ligands of some targets, as a whole, are likely to share common off-targets. Upon the term of ‘drug-target interactions’, we define such interactions between one target and a family of ligands for another target as ‘target-target interactions’.

**Figure 7:**
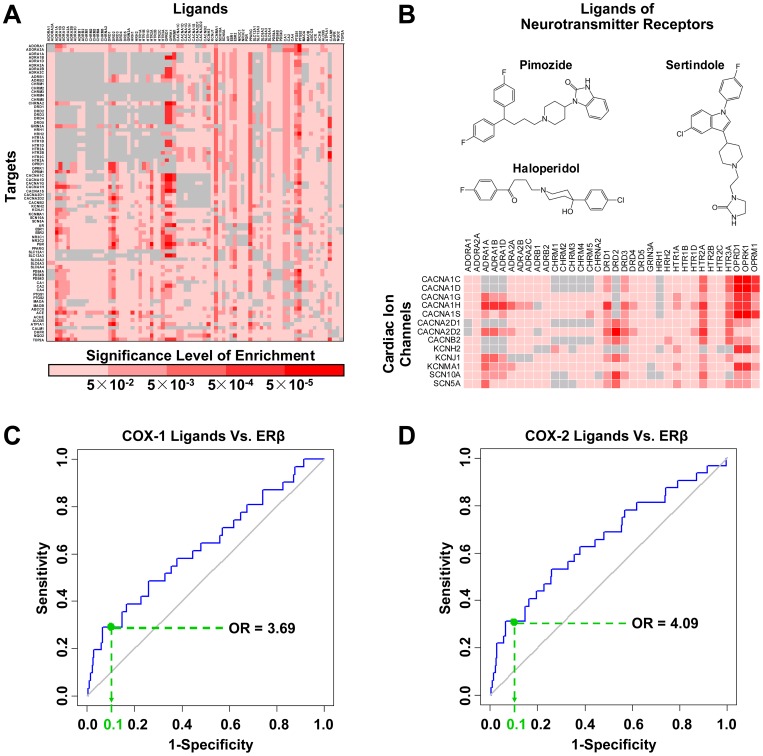
A summary of the highlighted potential target-target interactions. (A) The profile of target-target interaction is visualized with a matrix, in which each row and column represents a target and a family of ligands, respectively. The color of each cell represents the significance level of related target-target interaction. If a pair of targets are known to share ligands in DrugBank, their interaction (grey colored) would not be considered. (B) Potential interactions are broadly observed between antipsychotic drugs and cardiac ion channels. (C) & (D) The ligands of COX-1 (p = 3.08×10^−3^) and COX-2 (p = 1.02×10^−3^) generally show high LOI to estrogen receptor beta (i.e. ERβ).

As a unique output of CMap based model, target-target interactions are broadly observed across the DrugBank targets (Figure 7A & Data S2), some of which are consistent with previous experimental and clinical evidences. So unlike sporadic drug-target interactions, the target-target interactions are not just proposing individual cases of drug promiscuity, but providing explanations as to complicated actions that prevail in a family of drugs.

For instance, the designated ligands of neurotransmitter receptors generally showed high LOI to the cardiac ion channels (Figure 7B). Cardiac ion channels, present in the membranes of cardiac cells, control the movement of ions across membranes and determine the rate of heartbeat. Modification of these ion channels by drugs can bring about fatal arrhythmias [31]. On the other hand, the major ligands of neurotransmitter receptors are antipsychotic drugs, which are intended to selectively act on central nervous systems. However, as a whole, antipsychotics showed profound association with risk of arrhythmias [32]. Although previous studies have found a few direct interactions between individual antipsychotics (e.g. pimozide, haloperidol and sertindole etc.) and several ion channels [33]–[35], the mechanisms for antipsychotics induced cardiotoxicity remain unclear. Such target-target interactions in CMap suggest that the promiscuous interactions may not be limited to only a handful of antipsychotics and ion channels, but prevalent across many of them. In a systematic view [36], even moderate disturbance to multiple ion channels can add up to fatal impact, while it can be hardly explained by any single ion channel. Therefore, to understand the detailed mechanisms of drug induced cardiotoxicity, the binding affinities towards a variety of cardiac ion channels are recommended to be addressed.

Another example is the target-target interactions concerning with cyclooxygenases (PTGS1 and PTGS2, also known as COX-1 and COX-2). The ligands of cyclooxygenases are largely nonsteroidal anti-inflammatory drugs (NSAIDs), which are expected to relieve inflammation and pain. On the other hand, the NSAIDs are surprisingly reported to reduce cancer risk, by indirectly influencing carcinogenesis pathways [37]. However, the NSAIDs exhibit high LOI to estrogen receptors (targets for breast cancer drugs) in CMap based models (Figure 7C and Figure S3), suggesting that the anti-cancer activity of NSAIDs may also be attributed to direct interactions with anti-cancer drug targets. Consistent with our discovery, a recent study has initiatively identified an NSAID (i.e. diclofenac) targeting estrogen receptors [15]. Thus, we expect more hidden ligands for estrogen receptors to be found from NSAIDs, leading to a new prospective of anti-cancer drug development.

### Major discoveries and further efforts

In the present study, several important facts are initiatively discovered. First of all, we demonstrate that drug-induced gene-expression changes are directly correlated with ligand binding, and can be used solely to predict drug target. By adjusting the batch variation, CMap expression similarity can be used as the only indicator of DTI, which provides another cost-effective way of off-target identification. We therefore developed a prediction model, based only on gene-expression profiles. This model is suitable for those studies based only on limited information, such as the studies without large-scale gene network or expensive animal model.

Secondly, we find that not all targets are equally characterized in CMap, i.e. ligands binding to different targets would disturb the expression of different genes. Thus, unlike many one-size-fit-all methods interested in predicting all kinds of DTI, we prefer the target-specific models based on benchmark ligands. Especially for a series of well characterized targets, the ligands are proved to be highly predictable.

Finally, besides proposing sporadic hidden ligands or off-targets, researchers are paying more attention to integration of groups of drugs. For example, Iorio et al. [24] have integrated drugs into communities with similar mode of action, so as to find drugs acting on unexpected pathways. Similarly, we used CMap based model to specifically identify collective interactions between multiple drugs and targets (i.e. target-target interactions). This can help explain the reactions of not individual drugs but drug families, and increase the productivity of studies on drug repositioning and side effects [38].

Meanwhile, we are acutely aware that more effort should be made by learning from other CMap based studies related to the DTI issue. Primarily, DTI discovery is a very complicated problem that requires analyses of various types of information. In a recent study, Iskar et al. [39] have successfully identified a series of transcriptional modules by combining CMap with microarray data of rat models. These transcriptional modules then contribute to a better understanding of drug repositioning and identification of therapeutic targets. Following this example, we plan to further combine our model with other drug-related information (e.g. chemical-protein interactome [8], [40]), thus improving the power of CMap and the efficiency of DTI prediction. In addition, although our current work is focused on drug-target binding, it is well known that the impact of DTI has to be carried out through downstream biological pathways. As an example, Iorio et al. [24] have used CMap expression profiles to identify drugs acting on unexpected pathways. Enlightened by this study, we would extend our target prediction model to the level of downstream pathways, so as to better understand the biological implications of off-targets.

Taken together, we expect our model, along with other related works, can provide a full range of solutions to transcriptomic data analysis for researchers with different interests. By activating CMap and other transcriptomic data sources, gene-expression information would be readily integrated into DTI discovery pipelines in subsequent studies.

## Methods

### Normalization of batch variation in CMap expression profiles

The raw data of expression change fold in CMap is downloaded from CMap website (http://www.broadinstitute.org/cmap/). Suppose two different batches (say batch A and B) have n (n>0) pairs of instances treated by the same drug (i.e. bridges). For one certain gene, the expression change fold in the i-th bridge is designated as E(A,i) and E(B,i) in two batches, respectively. Taking all n pairs into consideration, we calculated the average variation of gene-expression profile between two batches in the logarithm form as
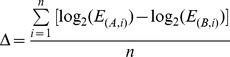



According to this quantified variation value, the expression of all instances (not limited to bridge instances) in batch B are transferred into
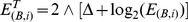
which approximates the change fold as if the instances are derived from cell cultures in batch A. And for all the 22,283 genes quantified by CMap microarray platform, the batch variation is bridged one gene after another, following the above procedures. As two different batches are merged into one, the merged new batch is again bridged with other batches and so on, until all CMap batches are adjusted. The data after adjustment as well as the R code can be downloaded at http://cpi.bio-x.cn/cmap/adjusted.zip

### BAES score calculation

By merging batches and combining instances treated by the same compound, we obtained a synthetic expression profile for each of the CMap compounds. The 22,283 genes are then ranked by fold change, that the most up-regulated genes are ranked at top and down-regulated at bottom. Every compound is in turn selected as reference, whose top and bottom ranked 250 genes are used as signature to query all compounds by GSEA algorithm. A pair of drugs, say drug A and B, could have two similarity scores, one score by querying B with A's signature and the other score in opposite. The BAES is defined as the average value of these two scores. Following the same procedure, we also calculated the similarity score with the original unadjusted CMap data to make a comparison.

### Naïve model measuring the likelihood of potential drug-target interactions

Given the direct correlation between drug-target binding and BAES score, we assume that the likelihood of a candidate compound (symbolized as *C*) binding to a specific target (symbolized as *T*) can be reflected by the overall expression similarity between the compound *C* and designated ligands of the target *T*. Suppose target *T* has N ligands enrolled in CMap, the likelihood of C interacting with T is estimated as follows
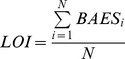
where *BAES_i_* represents the BAES score between the compound *C* and the i-th designated ligand of target *T*.

### Performance of leave-one-out cross validation

We perform leave-one-out cross validation to evaluate how well each target is characterized by CMap. Each CMap compound, including benchmark ligands and background drugs, serves as test set in turn, and all other compounds as training set. The LOI of the compound in test set is determined by its average BAES score to the benchmark ligands in training set. Then the benchmark ligands (positive compounds) and other CMap compounds (negative compounds) are classified by LOI, whose performance is illustrated with ROC curve. The 95% confidence interval of area under ROC curve is computed by the pROC package [26] for R environment (http://www.r-project.org/), with 2000 replicates of bootstrap test. The LOI corresponding to 90 percent specificity is set as the threshold to discriminate positive and negative compounds. The enrichment for benchmark ligands above threshold is calculated as an odds ratio (OR):
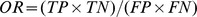
in which TP, TN, FP and FN represent true positive, true negative, false positive and false negative samples, respectively. To assess the statistical significance of enrichment, we performed Fisher's exact test based on the 2 by 2 contingency table corresponding to the four factors of odds ratio. To ensure the efficiency of bootstrapping and statistical test, the evaluation is confined to a total of 78 DrugBank human protein targets with at least 5 designated ligands enrolled in CMap.

## Supporting Information

Figure S1 BAES and DIPS for drug pairs sharing ATC code.
(DOC)

Figure S2 (A) HTR1A and HTR1B corresponds to 23 and 10 ligands enrolled in CMap, respectively. As 9 ligands are shared by both targets, the HTR1B ligands can almost be regarded as a subset of HTR1A ligands. (B) HTR1B shows not only better area under ROC curve (AUC), but also better enrichment odds ratio (OR) than HTR1A. However, due to the limited number of designated ligands for HTR1B, the statistical power of Fisher's exact test is impaired and the significance of enrichment could not be confirmed.
(DOC)

Figure S3 Besides estrogen receptor beta, the ligands of COX-1 (A) and COX-2 (B) also show significantly high LOI to estrogen receptor alpha (i.e. ERα), although ERα has not been confirmed as a well characterized target in CMap.
(DOC)

Table S1 The comparison between BAES and DIPS.
(DOC)

Table S2 The 302 CMap batches are merged in 6 stages.
(DOC)

Table S3 The bridge drugs shared by 10 big batches. If a bridge drug is used in multiple treatments (e.g. tanespimycin corresponds to 4 instances in all 10 batches), all possible instances-instance pairs are used as bridge to calculation batch variation.
(DOC)

Text S1 The difference between BAES and DIPS.
(DOC)

Text S2 The scenario of CMap batch bridging.
(DOC)

Data S1 Cross validation performance of individual targets.
(XLS)

Data S2 Target-target interactions measured by the significance level of ligands enrichment. Target pairs known to share ligands in DrugBank are marked with dash (–)
(XLS)
