# Correction: Prediction of Drug-Target Interactions for Drug Repositioning Only Based on Genomic Expression Similarity

**DOI:** 10.1371/annotation/958d4c23-4f1e-4579-b6ef-8ae1f828b1dd

**Published:** 2013-11-21

**Authors:** Kejian Wang, Jiazhi Sun, Shufeng Zhou, Chunling Wan, Shengying Qin, Can Li, Lin He, Lun Yang

The competing interests for this manuscript were missing. They should read "Lun Yang is an employee of GlaxoSmithKline, Philadelphia, Pennsylvania, United States of America and was previously employed by the Shanghai Jiao Tong University. This work was completed during his employment there and thus has no interest conflict with this company. The opinions and information in this article do not necessarily reflect the views and policies of the GlaxoSmithKline."

