# Supplementary figures and images for: Prediction of Drug-Target Interactions for Drug Repositioning Only Based on Genomic Expression Similarity
Source: PLoS Comput Biol. 2013 Nov 7;9(11):e1003315. doi: 10.1371/journal.pcbi.1003315 (PMC3820513; doi:10.1371/journal.pcbi.1003315)

**
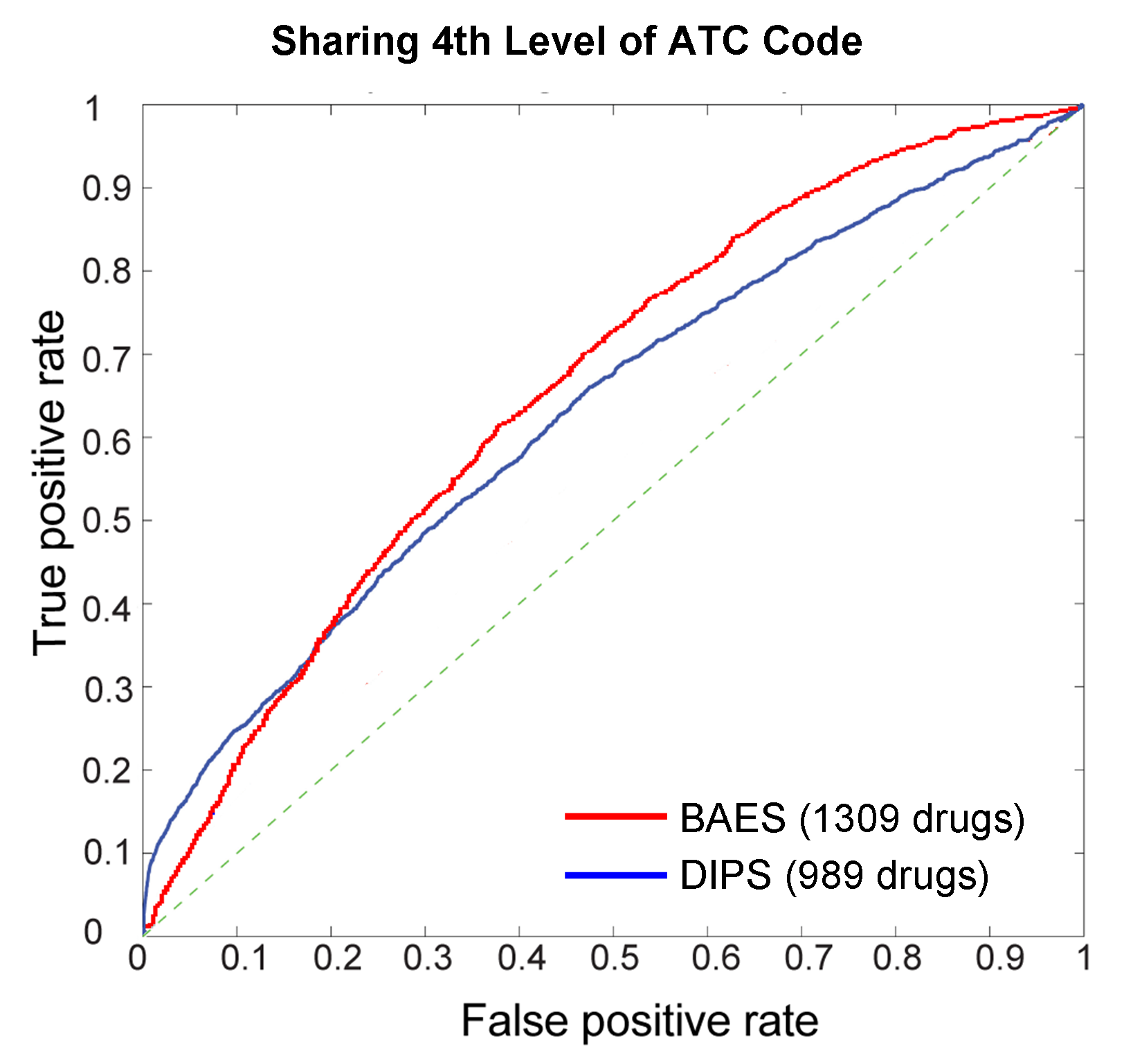
**

**Figure S1.** BAES and DIPS for drug pairs sharing ATC code.

Supplement: Figure S1 — BAES and DIPS for drug pairs sharing ATC code. (DOC) [file pcbi.1003315.s001.doc]
