# Supplementary material for: Prediction of Drug-Target Interactions for Drug Repositioning Only Based on Genomic Expression Similarity
Source: PLoS Comput Biol. 2013 Nov 7;9(11):e1003315. doi: 10.1371/journal.pcbi.1003315 (PMC3820513; doi:10.1371/journal.pcbi.1003315)
Supplement: Figure S2 — (A) HTR1A and HTR1B corresponds to 23 and 10 ligands enrolled in CMap, respectively. As 9 ligands are shared by both targets, the HTR1B ligands can almost be regarded as a subset of HTR1A ligands. (B) HTR1B shows not only better area under ROC curve (AUC), but also better enrichment odds ratio (OR) than HTR1A. However, due to the limited number of designated ligands for HTR1B, the statistical power of Fisher's exact test is impaired and the significance of enrichment could not be confirmed. (DOC) [file pcbi.1003315.s002.doc]

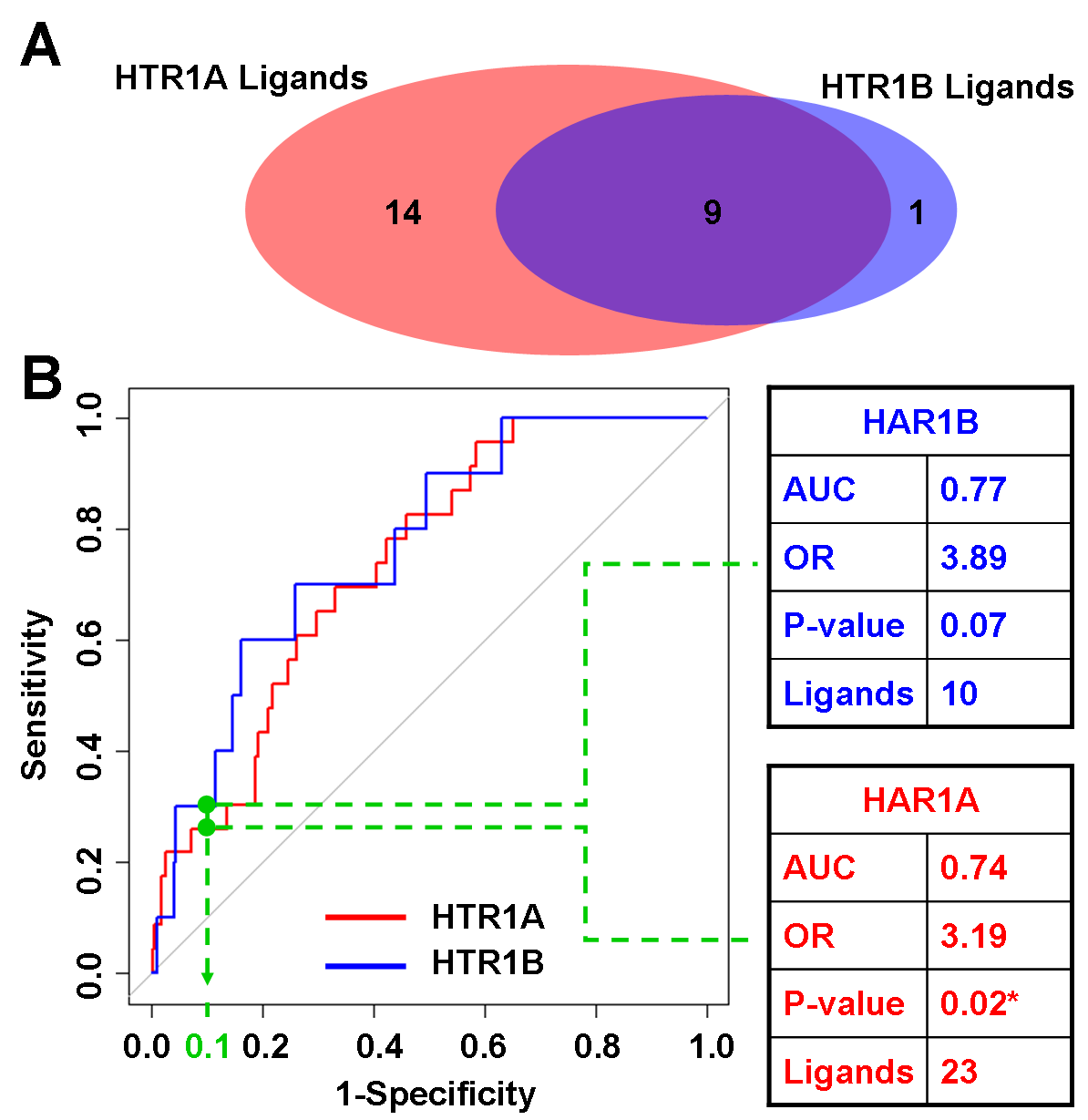


**Figure S2.** (A) HTR1A and HTR1B corresponds to 23 and 10 ligands enrolled in CMap, respectively. As 9 ligands are shared by both targets, the HTR1B ligands can almost be regarded as a subset of HTR1A ligands. (B) HTR1B shows not only better area under ROC curve (AUC), but also better enrichment odds ratio (OR) than HTR1A. However, due to the limited number of designated ligands for HTR1B, the statistical power of Fisher’s exact test is impaired and the significance of enrichment could not be confirmed.
