# Supplementary material for: Prediction of Drug-Target Interactions for Drug Repositioning Only Based on Genomic Expression Similarity
Source: PLoS Comput Biol. 2013 Nov 7;9(11):e1003315. doi: 10.1371/journal.pcbi.1003315 (PMC3820513; doi:10.1371/journal.pcbi.1003315)
Supplement: Figure S3 — Besides estrogen receptor beta, the ligands of COX-1 (A) and COX-2 (B) also show significantly high LOI to estrogen receptor alpha (i.e. ERα), although ERα has not been confirmed as a well characterized target in CMap. (DOC) [file pcbi.1003315.s003.doc]

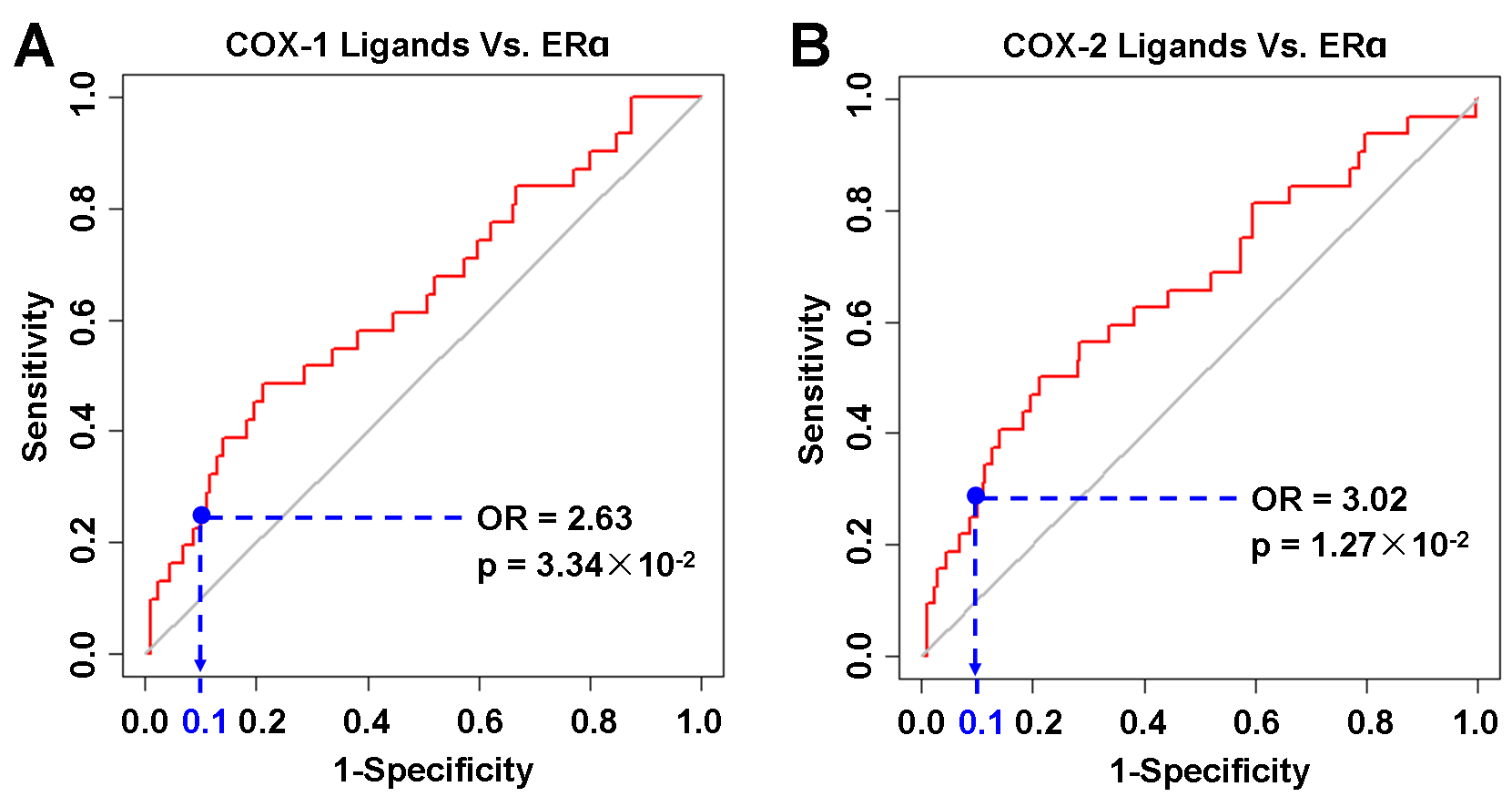


**Figure S3.** Besides estrogen receptor beta, the ligands of COX-1 (A) and COX-2 (B) also show significantly high LOI to estrogen receptor alpha (i.e. ERα), although ERα has not been confirmed as a well characterized target in CMap.
