# Supplementary material for: Prediction of Drug-Target Interactions for Drug Repositioning Only Based on Genomic Expression Similarity
Source: PLoS Comput Biol. 2013 Nov 7;9(11):e1003315. doi: 10.1371/journal.pcbi.1003315 (PMC3820513; doi:10.1371/journal.pcbi.1003315)
Supplement: Table S1 — The comparison between BAES and DIPS. (DOC) [file pcbi.1003315.s004.doc]

**Table S1.** The comparison between BAES and DIPS.

| **Similarity Score** | BAES | DIPS |
| --- | --- | --- |
| **Batch Filtration** | | |
| **Array Type** | All types of platform | HT_HG-U133A platform were selected (for HL60 cell line, the HG-U133A platform was also included) |
| **Batch Volume** | All batches | Batches containing over 25 treatments |
| **Drug Selection** | | |
| **Criterion** | All drugs | Drugs treated in all 3 cell lines (MCF7, PC3 and HL60) |
| **Drug Coverage** | 1309 drugs (100%) | 989 drugs (76%) |
| **Drug-drug Connectivity Coverage** | 856,086 drug pairs (100%) | 488,566 drug pairs (57%) |
| **Drugs Sharing ATC Code** | | |
| **ATC Source** | DrugBank | Not mentioned |
| **AUC of ROC curve** | 0.66 | 0.63 |
