# Supplementary material for: Prediction of Drug-Target Interactions for Drug Repositioning Only Based on Genomic Expression Similarity
Source: PLoS Comput Biol. 2013 Nov 7;9(11):e1003315. doi: 10.1371/journal.pcbi.1003315 (PMC3820513; doi:10.1371/journal.pcbi.1003315)
Supplement: Table S2 — The 302 CMap batches are merged in 6 stages. (DOC) [file pcbi.1003315.s005.doc]

**Table S2.** The 302 CMap batches are merged in 6 stages.

| **Stage** | I | II | III | IV | V | VI |
| --- | --- | --- | --- | --- | --- | --- |
| **ID of Adjusted Batches** | 506, 602, 603, 626, 650, 725, 727, 750, 757, 765 | 2, 5, 13, 17, 19, 21, 23, 25, 28, 33, 35, 36, 37, 38, 40, 41, 42, 44, 45, 46, 53, 54, 55, 56, 60, 61, 62, 63, 65, 66, 68, 69, 70, 71, 73, 74, 75, 79, 95, 101, 109, 111, 116, 119, 120, 502, 504, 505, 513, 514, 610, 611, 612, 613, 614, 615, 616, 617, 618, 619, 622, 623, 627, 628, 629, 630, 631, 632, 633, 634, 635, 636, 637, 640, 641, 642, 644, 645, 646, 647, 648, 649, 651, 653, 654, 655, 656, 657, 658, 659, 660, 661, 662, 663, 664, 665, 670, 671, 672, 673, 676, 677, 678, 680, 681, 682, 683, 685, 686, 687, 688, 689, 690, 691, 692, 693, 694, 695, 698, 699, 700, 701, 702, 703, 704, 705, 706, 707, 708, 709, 710, 711, 712, 713, 714, 715, 718, 719, 720, 726, 728, 729, 730, 731, 732, 733, 734, 735, 736, 737, 738, 741, 743, 744, 745, 746, 747, 748, 749, 751, 752, 753, 754, 755, 756, 758, 761, 762, 764, 766, 767, 771, 772, 1000, 1001, 1002, 1003, 1004, 1005, 1006, 1007, 1008, 1009, 1010, 1011, 1012, 1013, 1014, 1015, 1030, 1031, 1032, 1033, 1043, 1048, 1049, 1050, 1051, 1052, 1053, 1059, 1061, 1062, 1064, 1065, 1071, 1073, 1076, 1077, 1079, 1080, 1081, 1082, 1083, 1090, 1091, 1094, 1095, 110b, 26b | 1, 6, 7, 8, 16, 18, 20, 24, 29, 31, 39, 43, 58, 67, 82, 90, 94, 96, 98, 103, 107, 108, 112, 117, 1020, 1021, 1022, 1023, 1034, 1036, 1038, 1040, 1041, 1044, 1045, 1046, 1047, 1054, 1055, 1057, 1058, 1066, 1067, 1068, 1069, 1074, 1075, 1078, 1084, 1085, 1086, 1087, 1088, 1089, 1092, 1093, 22a, 2a, 7a | 59, 86, 87, 1026, 1027, 1028, 1029, 1035 | 1016, 1017, 1019 | 1024, 1025 |
