# Supplementary material for: Prediction of Drug-Target Interactions for Drug Repositioning Only Based on Genomic Expression Similarity
Source: PLoS Comput Biol. 2013 Nov 7;9(11):e1003315. doi: 10.1371/journal.pcbi.1003315 (PMC3820513; doi:10.1371/journal.pcbi.1003315)
Supplement: Table S3 — The bridge drugs shared by 10 big batches. If a bridge drug is used in multiple treatments (e.g. tanespimycin corresponds to 4 instances in all 10 batches), all possible instances-instance pairs are used as bridge to calculation batch variation. (DOC) [file pcbi.1003315.s006.doc]

**Table S3.** The bridge drugs shared by 10 big batches. If a bridge drug is used in multiple treatments (e.g. tanespimycin corresponds to 4 instances in all 10 batches), all possible instances-instance pairs are used as bridge to calculation batch variation.

| **Drug** | **Count of Instance** |
| --- | --- |
| ly-294002 | 4 |
| tanespimycin | 4 |
| valproic acid | 4 |
| haloperidol | 2 |
| trichostatin a | 2 |
| 15-delta prostaglandin j2 | 1 |
| chlorpromazine | 1 |
| acetylsalicylic acid | 1 |
| rosiglitazone | 1 |
| tretinoin | 1 |
| troglitazone | 1 |
| monorden | 1 |
| alpha-estradiol | 1 |
| masoprocol | 1 |
| fluphenazine | 1 |
| thioridazine | 1 |
| wortmannin | 1 |
| genistein | 1 |
| vorinostat | 1 |
| clozapine | 1 |
| prochlorperazine | 1 |
| alvespimycin | 1 |
| trifluoperazine | 1 |
