# Supplementary material for: Prediction of Drug-Target Interactions for Drug Repositioning Only Based on Genomic Expression Similarity
Source: PLoS Comput Biol. 2013 Nov 7;9(11):e1003315. doi: 10.1371/journal.pcbi.1003315 (PMC3820513; doi:10.1371/journal.pcbi.1003315)
Supplement: Text S1 — The difference between BAES and DIPS. (DOC) [file pcbi.1003315.s007.doc]

**The difference between BAES and DIPS**

Iskar et al. use the ‘mean-centering’ algorithm to remedy batch effect of CMap and measure expression similarity by the drug-induced gene-expression profile similarity (DIPS) score. We compared BAES with DIPS about scoring drugs sharing 4^th^ level of ATC code (Figure S1). DIPS shows better performance in early retrieval stage of ROC curve, but the treatment instances in CMap are so strictly filtered in multiple steps that a sizable proportion of information is lost. On the other hand, BAES exhibited better overall AUC and full coverage of all CMap instances (Table S1).

In summary, DIPS addressed a refined subset of CMap data with strong signal. By contrast, BAES provided an overall improvement for the entire CMap. The full coverage of CMap information enabled us to build an intact picture of promiscuity target pairs. Moreover, complex filtering steps are not necessary for BAES calculation, which would make BAES algorithm much easier to be further applied in other genomic expression data (e.g. Gene Expression Omnibus). Thus, we believe BAES and DIPS would be complementary to each other on the issue of batch effect adjustment.
