# Supplementary material for: Prediction of Drug-Target Interactions for Drug Repositioning Only Based on Genomic Expression Similarity
Source: PLoS Comput Biol. 2013 Nov 7;9(11):e1003315. doi: 10.1371/journal.pcbi.1003315 (PMC3820513; doi:10.1371/journal.pcbi.1003315)
Supplement: Text S2 — The scenario of CMap batch bridging. (DOC) [file pcbi.1003315.s008.doc]

**The scenario of CMap batch bridging**

In our scenario of batch effect adjustment, if a pair of instances from different batches is treated by the same compound, the corresponding batches would be bridged. If there are multiple bridges between two batches, all of them are used to evaluate the batch variation. All the CMap batches are merged together in 6 stages (Table S2). In stage I, 10 batches with more than 40 instances are merged through 23 bridge drugs (Table S3). Then in stage II, 220 batches bridged with the previous 10 batches are further merged. More batches in stage III are merged if only they are bridged with stage II batches, and so on. Finally, as no one batch is completely isolated from others, all 302 batches are successfully merged into one.
